# Vaccination of Heifers with Anaflatoxin Improves the Reduction of Aflatoxin B_1_ Carry Over in Milk of Lactating Dairy Cows

**DOI:** 10.1371/journal.pone.0094440

**Published:** 2014-04-08

**Authors:** Laura Giovati, Antonio Gallo, Francesco Masoero, Carla Cerioli, Tecla Ciociola, Stefania Conti, Walter Magliani, Luciano Polonelli

**Affiliations:** 1 Unità di Microbiologia e Virologia, Dipartimento di Scienze Biomediche, Biotecnologiche e Traslazionali, Università degli Studi, Parma, Italy; 2 Istituto di Scienze degli Alimenti e della Nutrizione, Facoltà di Agraria, Università Cattolica del Sacro Cuore, Piacenza, Italy; The University of Melbourne, Australia

## Abstract

It was previously reported that injection of anaflatoxin B_1_ (AnAFB_1_) conjugated to keyhole limpet hemocyanin (KLH), together with Freund's adjuvant, was effective in inducing in cows a long lasting titer of anti-aflatoxin B_1_ (AFB_1_) antibodies (Abs), cross-reacting with other aflatoxins, which were able to hinder, proportionally to their titer, the secretion of aflatoxin M_1_ (AFM_1_) into the milk of cows continuously fed with AFB_1_. According to anti-AFB_1_ Ab titer, 50% of the vaccinated cows were recognized as high responder animals. In an attempt to prepare a more effective formulation for vaccination of cows, it was compared the immunogenicity, in Holstein Friesian heifers, of AnAFB_1_ covalently conjugated to KLH or to recombinant diphtheria toxin (CRM_197_) molecules, and injected together with various adjuvants. This study demonstrated that injection of AnAFB_1_ conjugated to KLH and mixed with complete (priming) and incomplete Freund's adjuvant (boosters), as in the previous schedule of immunization, was the most effective regimen for inducing Ab responses against AFB_1_, although pre-calving administration could increase the effectiveness of vaccination, resulting in 100% high responder animals. After one booster dose at the beginning of the milk production cycle, anti-AFB_1_ Ab titers were comparable to those recorded at the end of the immunization schedule, and proved to be effective in reducing significantly AFB_1_ carry over, as AFM_1_, from feed to milk. Pre-calving vaccination of dairy heifers with conjugated AnAFB_1_, adjuvated with complete and incomplete Freund's adjuvant, may represent the most effective tool for preventing the public health hazard constituted by milk and cheese contaminated with aflatoxins.

## Introduction

Aflatoxins (AFs) are a group of mycotoxins produced mainly by strains of *Aspergillus flavus* and *A. parasiticus*. Over 20 AFs and derivatives have been isolated, but the major natural occurring AFs of fungal origin are B_1_, B_2_, G_1_, and G_2_, the B_1_ (AFB_1_) being the most important compound with respect to both prevalence and toxicity for man and animals [1].

Even if cases of acute intoxication may also occur, chronic toxicity of AFs is the most serious concern due to the estimated size of population at risk and the detrimental health effects associated with chronic aflatoxicosis [2], [3]. Chronic consumption of AFs has been identified as one of the major risk factors for the development of hepatocellular carcinoma and AFs are classified as Group 1 carcinogens by International Agency for Research on Cancer [2]. Furthermore, evidence suggests a relation between chronic AFs exposure and malnutrition, impaired growth, immunosuppression, and, consequently, susceptibility to infectious diseases [3].

In the liver, part of ingested AFB_1_ is biotransformed into the hydroxy derivative M_1_ (AFM_1_), which is then excreted into the milk of lactating mammals, including dairy animals [4]. As a result, AFB_1_ can pass through the food chain from animal feeds into milk as AFM_1_ (carry over). The occurrence of AFM_1_ in milk and its derivatives is a serious problem of food safety, and many countries defined specific limits for AFM_1_ in the milk and for AFB_1_ in the feed of dairy animals [5]. In spite of the strict selection of raw materials used for the manufacturing of feeds, production of AFM_1_-free milk is not always achieved and many surveys reported high levels of contamination of milk and milk derived food for humans and infants [6]–[9]. Furthermore, concern has been raised for the possible adverse cumulative effects of low amounts of AFM_1_, which could be present beneath the legal limit [10]. Physical, chemical and biological methods which have been tried for complete detoxification of AFM_1_ don't fulfill the efficacy, safety, and cost requisites of the task [11]. Addition to feeds of sequestering agents, able to bind selectively AFs, does not guarantee a complete prevention of AFs absorption in the gastro-intestinal tract, and some of them have been suspected to interfere with the assumption of micronutrients [12]–[14].

It was previously described an alternative prophylactic approach relying on an experimental vaccine based on the immunogen anaflatoxin B_1_ (AnAFB_1_), a non-toxic and non-mutagenic chemically modified preparation of AFB_1_, coupled to keyhole limpet hemocyanin (KLH) as carrier [15]. AnAFB_1_-KLH, administered with complete (priming) and incomplete Freund's adjuvant (boosters), was effective in inducing in cows a long lasting titer of anti-AFB_1_ antibodies (Abs) which were able to hinder the carry over of AFB_1_ as AFM_1_ into the milk of cows continuously fed with AFB_1_, without interfering with intradermal tuberculin test results. In particular, 50% of the vaccinated cows were recognized as low responder and 50% as high responder animals. For the latter, up to 46% reduction of average AFM_1_ in milk was recorded, as compared to animals immunized with KLH [15]. Whether AnAFB_1_-KLH conjugates would lose immunogenicity or be equally or more immunogenic when combined with other adjuvants was untested. Effect of conjugation of AnAFB_1_ with other protein carriers and effect of variations in the vaccination schedule on the immune response to AnAFB_1_ were unknown as well.

The aim of this work was to comparatively study the AFB_1_-specific Ab response in Holstein Freisian heifers immunized systemically with AnAFB_1_ conjugated with KLH and CRM_197_ carrier proteins and administered with Freund's adjuvant or aluminium hydroxide gel as immunological adjuvants, in order to select the most effective anti-AFB_1_ vaccine to further reduce AFM_1_ transfer in milk of cows exposed to AFB_1_.

## Materials and Methods

### Ethics statement

The research protocol and animal care were in accordance with the EC Council Directive guidelines for animals used for experimental and other scientific purposes [16].

The study has been approved by the local health authority “Azienda Unità Sanitaria Locale di Piacenza” (protocol number 50226) and by the National Ministry of Health according to legislative decree 116/92.

### Preparation of protein conjugates of AnAFB_1_

The immunogen AnAFB_1_, prepared converting AFB_1_ (Sigma-Aldrich, St. Louis, MO, USA) to AFB_1_-1(O-carboxymethyl) oxime using the method of Chu et al. [17], as previously described [15], was conjugated to KLH (Sigma-Aldrich) or CRM_197_ (List Biological Laboratories Inc., Campbell, CA, USA) to be used as immunogen. The coupling reaction to KLH was carried out using a method previously described [18]. N-hydroxysuccinimide (0.047 mmol) and N,N'-diisopropylcarbodiimide (9.3 μmol), dissolved in 600 μl of dimethylformamide (DMF), were added to AnAFB_1_ (5.19 μmol) in 600 μl of dry dichloromethane at 0°C, followed by 4-(dimethylamino)pyridine (0.041 mmol). Then, the active ester was slowly added to a pre-cooled aqueous buffered solution (31 mM Na_2_HPO_4_, pH 9.1) containing 20 mg KLH and not more than 10% (v/v) DMF. The mixture was kept at 4°C overnight, and then the conjugate was separated from unreacted reagents and by-products, desalted and extensively dialyzed against PBS by using a 10 kDa cut-off centrifugal filter tube (Microcon YM-10, Millipore Corporation, Bedford, MA, USA).

The water soluble carbodiimide method was used to conjugate AnAFB_1_ to CRM_197_ [19]–[22]. Briefly, AnAFB_1_ (1.6 μmol), dissolved in 31 μl of dimethyl sulfoxide, and CRM_197_ (0.16 μmol) were mixed in 1 ml of conjugation buffer (0.1 M 2-[N-morpholino] ethane sulfonic acid, 0.5 M NaCl, pH 5.0). Immediately prior to use, 10 mg of 1-ethyl-3-(3-dimethyl aminopropyl) carbodiimide hydrochloride (EDC) and 25 mg N-hydroxysulfosuccinimide (sulfo-NHS) were dissolved in 1 ml cold deionized water, and 500 μl EDC/sulfo-NHS solution were added to the reaction mixture. The reaction was continued for 2 h at room temperature with slow tilt rotation, after which hydroxylamine (final concentration 10 mM) was added to quench the reaction. The solution was incubated for 15 minutes at room temperature with slow tilt rotation. The conjugate was separated from unreacted reagents and by-products, desalted, and extensively dialyzed against PBS by centrifugation as described above.

The protein concentration was determined by using the Bradford method, using BSA (electrophoresis fractionated; Sigma-Aldrich) as external standard [23]. The amount of conjugated AnAFB_1_ was estimated based on the molar absorptivity of 20,950 (l•mol^−1^•cm^−1^) at 362 nm [24]. The ratio between the concentration of the bound toxin and that of the protein gave the loading degree of the conjugate. Two batches of the AnAFB_1_-KLH conjugate were produced at different times and were labelled as batch I and II. A loading degree of 6.4 and 8 mol AnAFB_1_:1 mol KLH was determined for batch I and II, respectively. A loading degree of 1 mol AnAFB_1_:1 mol CRM_197_ was determined. The conjugates were lyophilized and stored at −20°C before being used for immunization.

### Immunization of heifers with different vaccine formulations

Nineteen italian Holstein Friesian heifers (age of 18±1.5 months) housed at the CERZOO research and experimental center (San Bonico, Italy) were used for experimental immunization. The animals had free access to water and were fed *ad libitum*. The diet was composed by corn silage (408 g/kg), grass hay (408 g/kg), straw (102 g/kg), soybean meal (75 g/kg) and mineral-vitamin commercial mix (7 g/kg). On a dry matter basis (DM, 525 g/kg), the diet chemical composition was as follow: starch 264 g/kg DM, neutral detergent fiber 345 g/kg DM, and crude protein 165 g/kg DM.

The animals were regularly inspected by the veterinary services of the local health authority (Azienda Unità Sanitaria Locale). An official intradermal tuberculin test was carried out before starting and after the end of the vaccination schedule, and interpreted according to EC Commission Regulations [25]. The heifers were divided into groups of three or four and immunized at three weeks intervals with four doses (1 ml each) of one of six vaccine preparations: 1) 500 μg of batch I AnAFB_1_-KLH in complete (first dose) and incomplete (subsequent doses) Freund's adjuvant (Sigma-Aldrich) in a 1∶1 (vol/vol) ratio; 2) 500 μg of batch II AnAFB_1_-KLH in complete (first dose) and incomplete (subsequent doses) Freund's adjuvant in a 1∶1 (vol/vol) ratio; 3) 500 μg of batch II AnAFB_1_-KLH in incomplete Freund's adjuvant in a 1∶1 (vol/vol) ratio; 4) 500 μg of batch II AnAFB_1_-KLH in aluminium hydroxide gel (Sigma-Aldrich) in a 1∶1 (vol/vol) ratio; 5) 300 μg of AnAFB_1_-CRM_197_ in complete (first dose) and incomplete (subsequent doses) Freund's adjuvant in a 1∶1 (vol/vol) ratio; 6) 300 μg of AnAFB_1_-CRM_197_ in aluminium hydroxide gel in a 1∶9 (vol/vol) ratio. Animals of groups 1 and 2 received a fifth vaccine dose four months after calving, *i.e*. at the age of 31±1 months. Each vaccine preparation in Freund's adjuvant was administered intramuscularly in the neck, and each vaccine preparation in aluminium hydroxide gel was administered subcutaneously in front of the shoulder. The immunization schedule is summarized in Table 1. Animals of group 1 could be considered as a positive control of immunization as they received the same formulation previously described in lactating dairy cows [15].

**Table 1:** Immunization schedule.

Group	Antigen	Adjuvant	*N*	Route
1	AnAFB_1_-KLH, batch I	CFA (priming), IFA (boosters)	3	i.m.
2	AnAFB_1_- KLH, batch II	CFA (priming), IFA (boosters)	3	i.m.
3	AnAFB_1_-KLH, batch II	IFA	3	i.m.
4	AnAFB_1_-KLH, batch II	AlOH_3_	4	s.c.
5	AnAFB_1_-CRM_197_	CFA (priming), IFA(boosters)	3	i.m.
6	AnAFB_1_-CRM_197_	AlOH_3_	3	s.c.

CFA: complete Freund's adjuvant; IFA: incomplete Freund's adjuvant; AlOH_3_: aluminium hydroxide gel; i.m.: intramuscular; s.c.: subcutaneous; *N*: number of heifers per group. Immunization consisted of a priming dose (day 0) of 500 μg AnAFB_1_-KLH or 300 μg AnAFB_1_-CRM_197_ with adjuvant, as indicated, followed by three doses with the same amount of conjugate with adjuvant, as indicated, at intervals of three weeks. Animals of groups 1 and 2 received a fifth vaccine dose 4 months after calving, which was at age of 27±1.1 months. A loading degree of 6.4 and 8 mol AnAFB_1_:1 mol KLH was determined for batch I and II, respectively. A loading degree of 1 mol AnAFB_1_:1 mol CRM_197_ was determined.

Animals were bled via the jugular artery prior to each immunization and two weeks after last booster. Animals of groups 1 and 2 were bled at scheduled times thereafter. Blood was stored 60 min at 37°C to allow clotting, centrifuged (1500 *g*, 10 min, room temperature), and the serum fraction aspirated and stored at −20°C.

### Enzyme-linked immunosorbent assay (ELISA)

Detection and titration of specific anti-AFB_1_ Abs were carried out by ELISA, as previously described [15]. Briefly, serially ten-fold diluted (from 1∶4 and 1∶10 to 1∶400,000) immune serum or control (pre-immune serum) was added to each well of polystyrene microtiter plates coated with AFB_1_-BSA conjugate (Sigma-Aldrich) or BSA control protein. After incubation, reaction was detected by adding rabbit anti-bovine IgG (whole molecule) peroxidase conjugate Abs (Sigma-Aldrich, product number A7414) and chromogen/substrate solution. The optical density (OD) at 450 nm was read by using a Multiskan Ascent spectrophotometer (Labsystems, Helsinki, Finland), and the titer of each immune serum was defined as the inverse of the highest dilution that gave 0.1 OD above the pre-immune serum at the same dilution. To compensate for between-plate variability, individual plates were normalized to the mean of the appropriate positive control. For statistic evaluation the logarithms of anti-AFB_1_ Ab titers were considered.

### Treatment of dairy cows with AFB_1_

Two months after last vaccine dose, an AFB_1_ contaminated diet was administered to animals of group 1 (n = 3), group 2 (n = 3), and unvaccinated animals (control group, n = 6). On average, animals had an age of 33±1 months, 219.4±21 days in milk, and average milk yield of 28.7±6.3 kg/day/cow. Basal diet samples were collected on days 0 and 11 of the experimental period, dried at 55°C in a ventilated oven to constant weight, and then ground with a 1 mm sieve (Thomas-Wiley Laboratory Mill, Arthur H. Thomas Co., Philadelphia, PA, USA) and frozen until analysed for AFs. The AFB_1_ content in the basal diet, measured by HPLC-fluorescence after immunoaffinity separation, as previously described [15], was 0.13±0.01 μg/kg, corresponding to about 2.8±0.25 μg/cow/day based on an estimated ingestion of 21.5 kg/cow/day dry matter. On the days of experiment, animal were given via oral drench, before feeding, a bolus of naturally AFB_1_-contaminated corn meal in about 300 g of AFB_1_-free soy bean meal, giving a calculated daily AFB_1_ total ingestion of 102±0.1 μg. AFB_1_ contents of different supplied batches were tested and results demonstrated a good homogeneity. Considering the estimated average ingestion, a corresponding AFB_1_ contamination of 4.74 μg/kg of diet could be calculated, a value in the range commonly reported on standard field conditions [26], [27].

Experimental periods lasted 16 days, consisting of 11 days of intoxication and 5 days of clearance (no AFB_1_ in the diet). Individual milk samples were collected at day 0, 1, 3, 5, 7, 9, 11, 12, 14, and 16. A representative sample for each day of milking was obtained and stored at −18°C for subsequent analysis.

### Quantification of AFM_1_ in milk samples and carry over calculation

AFM_1_ was quantified by HPLC in milk samples defatted by centrifugation, filtered, and passed through an immunoaffinity column, as previously described [15].

The carry over rate of AFB_1_ in milk as AFM_1_ was calculated as the percentage ratio between the daily amount of AFM_1_ excreted in milk at the plateau condition and the daily amount of AFB_1_ ingested by the animals.

### Statistical analyses

All data were presented as means ± standard deviations (SD), and analysed as repeated measurements according to a completely randomized design. In particular, the statistical model adopted for data collected over time from different groups (anti-AFB_1_ Ab titers and AFM_1_ concentration at steady-state condition) included the fixed effects of treatment, time of measurement and treatment × time of measurement interaction. The steady-state condition of AFM_1_ was determined as suggested by Littell et al. [28]. To assess persistence of specific Abs over time, anti-AFB_1_ titers were analysed considering the fixed effect of time of measurement. Cows within groups were the subject of repeated measurements. Least square means (LSMeans) were post-hoc compared by using Tukey multiple comparison test. P values<0.05 were considered significant, while P between 0.05 and 0.10 were considered as a trend.

## Results

### Titration of anti-AFB_1_ Abs

The titers of specific anti-AFB_1_ Abs detected by ELISA in sera from individual heifers immunized either with AnAFB_1_-KLH or AnAFB_1_-CRM_197_ are shown in Table 2. For all of the heifers, pre-immune control sera showed negligible binding to AFB_1_-BSA. When sera were incubated with control BSA (unconjugated), only a very low degree of nonspecific binding was detected (data not shown). Heifers of groups 1 and 2, primed with batch I and II AnAFB_1_-KLH in complete Freund's adjuvant showed a titer of anti-AFB_1_ Abs of 10,000 after injection of the second dose in incomplete Freund's adjuvant. After the third dose only one heifer belonging to group 1 showed an Ab titer of 40,000. The fourth injection did not increase the Ab titer observed after the first three doses (P>0.05). Heifers of group 3, primed and boosted with batch II AnAFB_1_-KLH in incomplete Freund's adjuvant, reached a peak (titers of 400 or 1,000) after the second dose. The titers of two heifers declined thereafter. At week 11, after the administration of four doses of the vaccine, the average Ab titer of this group was lower than the ones of groups 1 and 2 (P<0.05). Immunization with 4 doses of batch II AnAFB_1_-KLH in alum induced no detectable anti-AFB_1_ Ab titers in any of the heifers of group 4 (data not shown). Priming with AnAFB_1_-CRM_197_ in complete Freund's adjuvant and injection of the second dose in incomplete Freund's adjuvant did induce anti-AFB_1_ Ab production in 3 out of 3 heifers of group 5. However, the average Ab titer of this group was not as high as those in AnAFB_1_-KLH immunized heifers of groups 1 and 2 (P<0.05). Administration of AnAFB_1_-CRM_197_ in alum induced anti-AFB_1_ Ab production in 2 out of 3 heifers of group 6. After the administration of four vaccine doses, average Ab titer of group 6 was comparable to that of group 3 (P>0.05) and lower than those of group 5 (P<0.05).

**Table 2:** Anti-AFB_1_ Ab titers in vaccinated heifers.

Group	Cow	Anti-AFB_1_ Ab titer				
		Week 0	Week 3	Week 6	Week 9	Week 11
**1**	415	0	3.00	4.00	4.60	4.60
	418	0	3.00	4.00	4.00	4.00
	422	0	2.60	4.00	4.00	4.00
	**Average**	**0±0**	**2.87±0.23**	**4.0±0**	**4.2±0.35**	**4.2±0.35**
**2**	412	0	3.60	4.00	4.00	4.00
	421	0	3.00	4.00	4.00	4.00
	425	0	3.60	4.00	4.00	4.00
	**Average**	**0±0**	**3.4±0**	**4.0±0**	**4.0±0**	**4.0±0**
**3**	432	0	2.60	2.60	2.00	0.00
	433	0	2.00	3.00	3.00	3.00
	434	0	2.00	2.60	2.00	2.00
	**Average**	**0±0**	**2.20±0.35**	**2.73±0.23**	**2.33±0.58**	**1.67±1.53**
**5**	410	0	0	2.00	3.60	3.00
	419	0	2.00	3.00	3.60	3.60
	428	0	0	3.00	4.00	4.00
	**Average**	**0±0**	**0.67±1.15**	**2.67±0.58**	**3.73±0.23**	**3.53±0.50**
**6**	411	0	0	0	0	0
	423	0	0	2.60	3.00	3.00
	426	0	2.60	3.00	3.00	3.00
	**Average**	**0±0**	**0.87±1.50**	**1.87±1.63**	**2.00±1.73**	**2.00±1.73**

Heifers of different groups were immunized according to the schedule reported in Table 1. For all the groups, booster injections were performed at week 3, 6, and 9. Ab titers were determined by the method described in the text. The titer of each immune serum was defined as the inverse of the highest dilution that gave 0.1 OD above the pre-immune serum at the same dilution and presented in table on a logarithmic scale for comparative purposes (undetectable anti-AFB_1_ Abs were assigned a value of 0). The fixed effects of ANOVA (i.e., week, group and their first order interaction) were significant at P<0.05 and comparisons of interest among means were described in the text.

In Table 3 is summarized the anti-AFB_1_ response among the vaccinated heifers in terms of high-responder (anti-AFB_1_ Ab titer ≥10,000), low-responder (titer between 1,000 and <10,000) and non-responder (titer<1,000) animals at the end of the vaccination schedule.

**Table 3:** Distribution of high, low and non-responders among vaccinated heifers.

	Anti-AFB_1_ Ab titer		
	Non-responder (titer <1,000)	Low-responder (titer 1,000-<10,000)	High-responder (titer ≥10,000)
Group 1 (n = 3)	-	-	3
Group 2 (n = 3)	-	-	3
Group 3 (n = 3)	2	1	-
Group 4 (n = 4)	4	-	-
Group 5 (n = 3)	-	2	1
Group 6 (n = 3)	1	2	-

At the end of the vaccination, cows of groups 1 and 2 presented no difference (group effect not significant) in anti-AFB_1_ Ab titers (P>0.05). Batch I and II vaccines were therefore considered as replicates of same treatment and the high responders animals of groups 1 and 2 were pooled for subsequent monitoring and carry over studies.

The vaccination regimen did not induce delayed hypersensitivity to *Mycobacterium tuberculosis* in any of the vaccinated heifers, as demonstrated by negative intradermal tuberculin test.

### Monitoring of anti-AFB_1_ Abs

**Figure 1:**
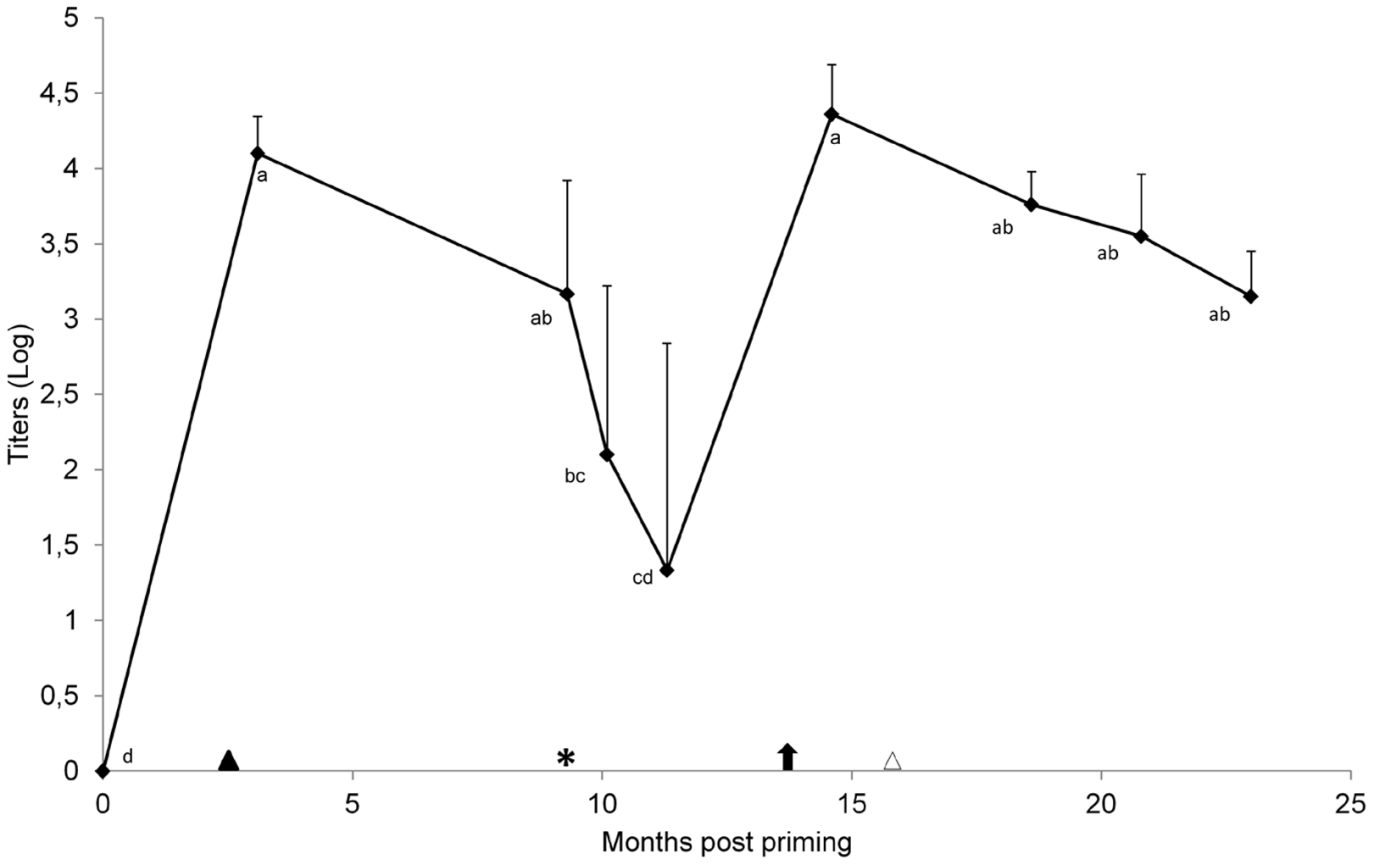
Monitoring of anti-AFB_1_ Ab titers. Mean anti-AFB_1_ Ab titers of high responder cows (n = 6) following vaccination with AnAFB_1_-KLH in complete (first dose) and incomplete (3 boosters at three weeks intervals) Freund's adjuvant. Two animals were removed from treated group (at 13.8 and 20.8 months post priming, respectively) for health injuries unrelated to vaccination. Partum was at an average of 9.6±0.75 months after first dose vaccine administration. Data are presented (on a logarithmic scale) as mean and SD. ▴, fourth dose of vaccine (2.1 months); 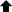, booster (13.8 months); 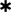, partum (9.6±0.75 months); ▵, AFB_1_ administration (15.6 months). Titers corresponding to points with different subscripts (a, b, c, d) differ significantly as *post-hoc* evaluation by Tukey's test (P<0.05).

Anti-AFB_1_ Ab titers of selected high responder animals were monitored at scheduled times to assess persistence of specific Abs over time and to evaluate the influence of pregnancy and partum on the immunological status of animals. As shown in Figure 1, anti-AFB_1_ Ab titers declined over time following vaccination. No difference (P>0.05) was observed between titers recorded in sera collected 15 days before the time of expected parturition and 15 day after partum, indicating that physical and metabolic stresses associated with late pregnancy, calving, early lactation, and peak milk yield had no effects on the titer of anti-AFB_1_ Abs.

After one booster dose at the beginning of the milk production cycle, anti-AFB_1_ Ab titers increased (P<0.05) with values ranging between 10,000 and 40,000 at 14.6 months post priming, the booster being effective to retrieve an Ab response against AFB_1_. Subsequently, titers declined over time, while administration of AFB_1_ in the diet had no effect on anti-AFB_1_ Ab decrease (P>0.05).

### Quantification of AFM_1_ in milk samples and carry over calculation

**Figure 2:**
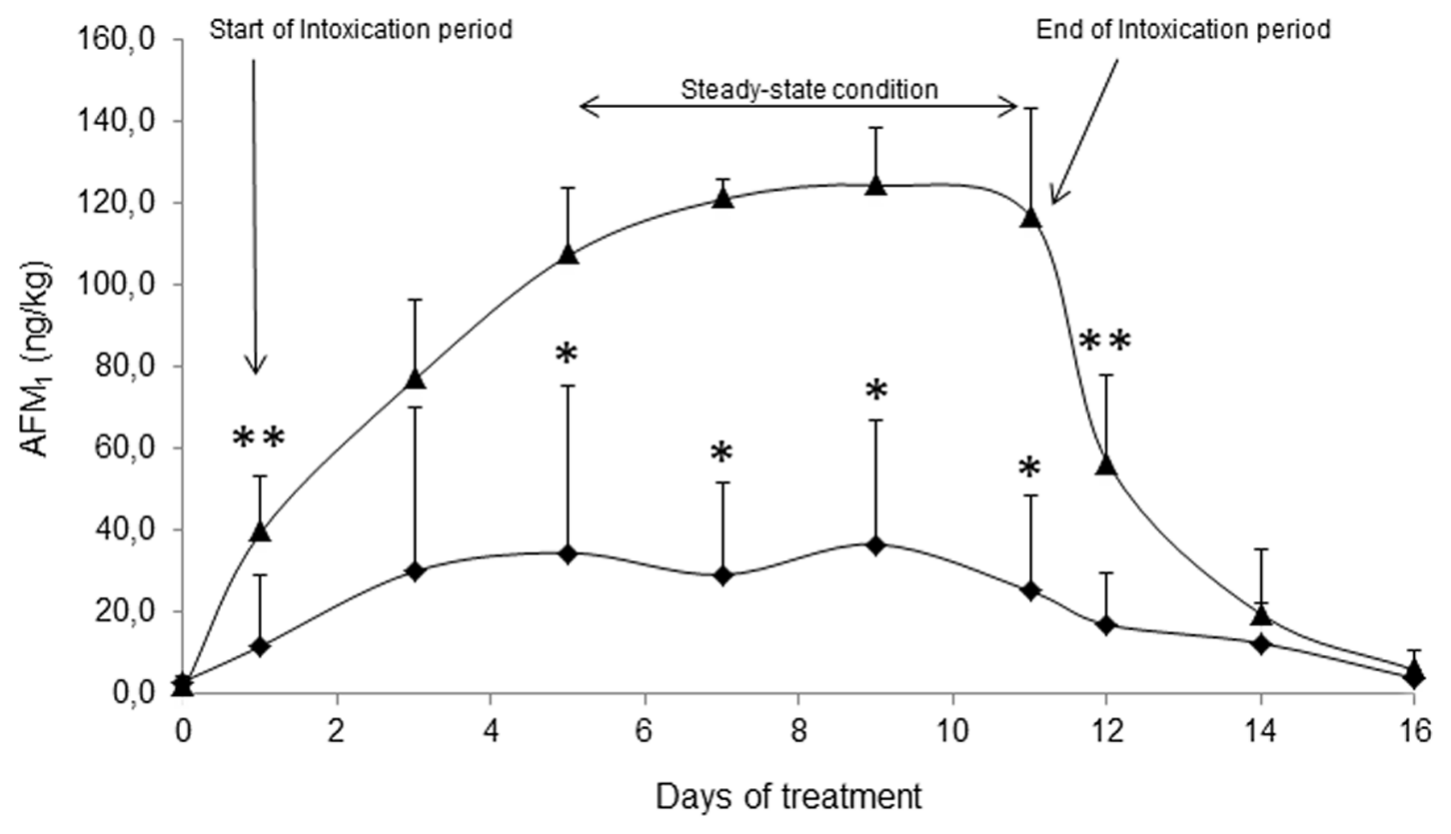
Average AFM_1_ concentration in milk. Six vaccinated (⧫) and six unvaccinated control (▴) cows were fed 102 μg of AFB_1_/day from day 1 to day 11. Data are presented as mean ± SD. Within each day, differences between vaccinated and control cows are marked (**P<0.10, *P<0.05).

The efficacy of anti-AFB_1_ Abs in reducing the carry over of AFB_1_ as AFM_1_ into milk was evaluated following parturition and starting of milk production by monitoring AFM_1_ concentrations in milk. Basal diet AFB_1_ level contributed to a milk AFM_1_ contamination of 2.63±1.11 ng/kg, as measured in milk sampled on day 0. Results of AFM_1_ quantification in the milk collected from cows vaccinated when heifers and control unvaccinated cows during the intoxication period (102 μg of AFB_1_ *per* cow *per* day) are shown in Figure 2. AFM_1_ concentration in milk increased at every milking and reached a steady-state condition from day 5 of intoxication period for both groups, showing a similar behavior among animals, the random cow effect not being significant. On day 11, when AFB_1_ administration was stopped, the mean AFM_1_ concentration decreased quickly to return at the base line on day 16. AFM_1_ concentrations recorded in the milk of control cows were consistent with carry over rates observed under field conditions [29].

At the steady state condition, the average AFM_1_ concentration in milk collected from vaccinated cows was 74% lower than in milk of control animals (P<0.05). Similarly, the carry over rate calculated in vaccinated cows (0.77%) was lower with respect to control animals (3.40%) (P<0.05) (Table 4).

**Table 4:** Average concentration of AFM_1_, total excretion of AFM_1_, carry over rate, and milk production traits.

	Controls	Vaccinated
AFM_1_ (ng/kg)	118±17	31±28
Total AFM_1_ (μg/cow/day)	3.47±0.70	0.79±0.58
Carry over (%)	3.40±3.82	0.77±0.57
Milk production (kg/day)	32.13±2.82	32.25±2.96
Milk fat (g/kg)	36.2±0.30	36.5±0.42
Milk crude protein (g/kg)	34.2±0.28	33.8±0.26

The values were calculated at the steady state condition (days 5, 7, 9 and 11) in groups of cows with average daily production of 28.7±6.3 kg of milk, subjected to ingestion of 102 μg AFB_1_/cow/day.

## Discussion

In a previous work it was reported an alternative approach for the prevention of AFB_1_ carry over as AFM_1_ in cow's milk, relying on an experimental KLH-conjugate vaccine based on the antigen AnAFB_1_ [15]. The vaccine was effective in inducing in cows long lasting anti-AFB_1_ Abs, and Ab responses were highly predictive of reduction of AFB_1_ carry over as AFM_1_ into the milk following ingestion of contaminated feed. On the basis of the anti-AFB_1_ Ab titer, cows were categorized into high responders (titers of 10,000 or higher) and low responders (titers between 1,000 and <10,000). As compared to unvaccinated controls, in high responders cows a reduction up to 46% of average AFM_1_ in milk was recorded [15].

The purpose of this work was to present further developments of this anti-AFB_1_ experimental vaccine, in order to obtain a more potent Ab response and then to increase prevention of AFB_1_ carry over as AFM_1_ from feed to milk in vaccinated cows.

Several factors, including the selection of the carrier protein and adjuvant are known to affect the immunogenicity of conjugate vaccines [30], [31]. To study the effect of the carrier protein on AnAFB_1_ immunogenicity, the antigen was covalently coupled to CRM_197_, a non-toxic mutant of diphtheria toxin [32] which demonstrated, in several vaccines, both safety and consistent Ab induction against the immunizing antigens [33]. AnAFB_1_-KLH and AnAFB_1_-CRM_197_ conjugates were formulated with incomplete Freund's adjuvant and aluminium hydroxide, two adjuvants approved for the development of commercial vaccines in veterinary medicine, as well as with complete Freund's adjuvant used for previous studies. The immunogenicity and protective performances against AFB_1_ carry over from feed to milk were compared after administration of the different vaccine formulations.

To study the effect of animal age on anti-AFB_1_ response, and assess the possibility to confer protection from the beginning of the milk production cycle, the vaccines were administered to young heifers before calving. In particular, 3 or 4 heifers were immunized for each vaccine preparation (Table 1). The number of animals could be considered quite small and this could represent a possible limit of current results. Anyway, the low intra-group Ab titers variability (Table 2) seemed to have not reduced the power of adopted tests to verify differences.

Immunogenicity of the AnAFB_1_ antigen either conjugated to KLH or CRM_197_ was affected by administration together with different adjuvants. In fact, while KLH was a more potent carrier than CRM_197_ in Freund's adjuvant, CRM_197_ was a more potent carrier than KLH in aluminium hydroxide. AnAFB_1_-KLH with complete (priming) and incomplete Freund's adjuvant (boosters) was the most effective formulation for inducing Ab responses against AFB_1_, and consistently high titers of Ab specific to AFB_1_ were induced in all of the heifers. Although Freund's complete adjuvant is known to produce a strong and long-lasting immunity to a broad range of antigens, concerns over severe injection-site reactions and its potential carcinogenicity hinder its use as an adjuvant for human vaccines, and Freund's complete adjuvant is not recommended for animal use in certain countries [34]. The use of Freund's complete adjuvant in the first dose of the vaccine proved to be necessary to obtain a potent anti-AFB_1_ response. On the other hand, after a single administration of AnAFB_1_-KLH in complete Freund's adjuvant and boosters in incomplete Freund's adjuvant no adverse effects on animal health and milk production traits were observed. Moreover, any interference with the primary diagnostic test for bovine tuberculosis was excluded.

The data obtained suggest that pre-calving administration can increase the effectiveness of vaccination, resulting in 100% high responder animals, while the same formulation administered in lactating dairy cows was previously shown to result in 50% high responder animals [15]. Moreover, anti-AFB_1_ Abs reached a peak after the third dose of vaccine.

Anti-AFB_1_ Ab titers of vaccinated heifers decreased during pregnancy and after calving but, after one booster dose at the beginning of the milk production cycle, titers returned to levels comparable to the ones obtained at the end of the immunization schedule. This finding suggests that an annual booster could be proposed to maintain a suitable protection. Anti-AFB_1_ Abs proved to be effective in reducing significantly AFB_1_ carry over as AFM_1_ in milk following ingestion of contaminated feed. While transfer of AFM_1_ in milk of control animals was consistent with carry over rates observed under field conditions, carry over of AFB_1_ as AFM_1_ was reduced to 0.77% in vaccinated heifers, resulting in a 74% reduction of AFM_1_ concentrations in milk.

Overall, the results of this study indicate that vaccination of heifers prior to calving could confer protection from AFB_1_ carry over as AFM_1_ in milk from the beginning of the milk production cycle. Easy detection of anti-AFB_1_ Abs could allow monitoring the immunological status of milk animals in order to determine the protective titer and evaluate the need for booster injections.

Reduction of AFM_1_ in milk obtained in vaccinated cows was notably higher than the one that can be obtained by inclusion in animal diet of sequestering agents, able to prevent AFs absorption in the gastro-intestinal tract [35], [36]. Addition of sequestering agents could reduce AFM_1_ transfer in the milk of no more than 50%, depending on the extent of contamination, animal species, site of AF absorption, method of addition to diets, and dose [26], [37]–[39].

Vaccination is economically sustainable, the resulting protection could persist over a whole milk production cycle, and alternative treatments, such as AFs sequestering agents, could be associated, in presence of high contamination of feed [15].

Vaccination can contribute significantly to the primary objective of minimizing population exposure to AFM_1_. Abs induced in cows after vaccination may in fact capture even small amounts of AFB_1_, preventing the presence of AFM_1_ in concentrations which, although lower than the legal limits, may determine problems related to chronic intake over time. Vaccination of dairy cattle could also ensure better health quality of milk and milk products when contamination cannot be maintained within acceptable limits by currently used methods.

Although some other factors, such as the fate of AFB_1_ captured by Abs or the consequences of accidental ingestion of very high amount of AFB_1_, should be further investigated, vaccination of dairy animals against AFB_1_ constitutes a feasible alternative measure which could significantly reduce the threat to human health posed by AFs.
